# Pressure gradient post-percutaneous coronary intervention: beyond angiography

**DOI:** 10.1007/s12471-022-01709-4

**Published:** 2022-07-05

**Authors:** C. K. M. Boerhout, J. J. Piek

**Affiliations:** Department of Clinical and Experimental Cardiology, Heart Centre, Amsterdam UMC—location AMC, Amsterdam, The Netherlands

The discordance between the angiographic and physiological characteristics of coronary artery disease (CAD) is increasingly recognised. In the early days of percutaneous coronary interventions, German-born physician-scientist Andreas Gruentzig was already aware of the need for measuring pressure gradients before and after balloon inflation in the diseased coronary artery [1]. However, in subsequent decades, the focus primarily shifted towards angiographic assessment alone.

Only in recent years have studies regarding the importance of functional assessment of CAD emerged, and clinical practice has slowly been adapting to a more physiology-guided approach. In this issue of the *Netherlands Heart Journal*, the article by Masdjedi et al. [2] importantly adds to the current literature with a post hoc analysis of the FFR-SEARCH registry regarding the prognostic value of the resting diastolic pressure ratio (dPR) post-percutaneous coronary intervention (PCI). In their study, they found that approximately 15% of the patients with optimal angiographic PCI results had an abnormal post-PCI dPR (< 0.89). Interestingly, these patients had a higher incidence of target vessel failure and a higher cardiac mortality rate.

An association between abnormal post-PCI pressure gradients and clinical outcomes was first described almost two decades ago by Pijls et al. [3]. Post-PCI fractional flow reserve (FFR) was a strong independent predictor of clinical outcomes. Since then, it is argued that maximal hyperaemia is essential in order to detect the smaller pressure gradients post-PCI, and the adoption of post-PCI physiological assessment is hampered by the increased procedural time and effort. However, the findings by Masdjedi et al. [2] show that the easily available resting dPR also has prognostic value in the assessment of post-PCI results. In this respect, it is important to mention some pathophysiological mechanisms that could explain the association between abnormal resting pressure gradients and clinical outcomes. First, PCI causes distal embolisation, resulting in a hyperaemic response and creating a gradient across residual stenosis post-PCI [4]. Secondly, diffuse atherosclerosis—which may be underestimated by angiographic assessment alone—creates a pressure drop along the diseased vessel. Especially in these cases, resting pressure gradient measurement has an advantage over the FFR with the possibility of pull-back measurements and regarding the interplay effect of sequential lesions [5].

Altogether, the importance of physiological assessment of post-PCI results is becoming increasingly clear. Masdjedi et al. [2] add to the evolving literature and importantly show that resting pressure ratios are also associated with impaired clinical prognosis. Hereby, the application of post-PCI assessment beyond angiographic results—with easily available resting pressure gradients or other non-hypaeremic modalities—is facilitated and enables future optimisation of PCI therapy.
